# Supplementary material for: Left-right asymmetric and smaller right habenula volume in major depressive disorder on high-resolution 7-T magnetic resonance imaging
Source: PLoS One. 2021 Aug 3;16(8):e0255459. doi: 10.1371/journal.pone.0255459 (PMC8330903; doi:10.1371/journal.pone.0255459)
Supplement: S1 Table — * indicates significant difference (P < 0.05). The statistical analysis was performed using a Student’s t-test. Abbreviations: HC, healthy control; MDD, major depressive disorder; SD, standard deviation. (DOCX) [file pone.0255459.s001.docx]

**S1 Table**. **Group differences in the volume and T1 value of the habenula segmented by both examiners** **between MDD and HC**

|  | Hemisphere | MDD (n = 33) | HC (n = 36) | t score | *P* values |
| --- | --- | --- | --- | --- | --- |
| Volume  (in mm^3^ ± SD) | Right | 16.43 ±3.24 | 18.54 ± 3.91 | −2.431 | 0.018^*^ |
|  | Left | 18.37 ± 3.39 | 18.47 ± 5.04 | −0.090 | 0.929 |
| T1  (value ± SD) | Right | 1200.96 ± 41.29 | 1207.84 ± 32.35 | −0.773 | 0.442 |
|  | Left | 1225.03 ± 34.81 | 1220.51 ± 31.90 | 0.564 | 0.575 |

^*^ indicates significant difference (*P* < 0.05).
The statistical analysis was performed using a Student’s t-test
Abbreviations: HC, healthy control; MDD, major depressive disorder; SD, standard deviation
