# Supplementary material for: Left-right asymmetric and smaller right habenula volume in major depressive disorder on high-resolution 7-T magnetic resonance imaging
Source: PLoS One. 2021 Aug 3;16(8):e0255459. doi: 10.1371/journal.pone.0255459 (PMC8330903; doi:10.1371/journal.pone.0255459)
Supplement: Attachment — Submitted filename: Response to reviewers_final version.docx [file pone.0255459.s002.docx]

**Responses to Reviewer #1:**
We thank the reviewer for the comments on our article. We believe that the article has been considerably improved based on the reviewer’s comments and suggestions. Please see below the individual responses to their comments.

***Comments to the Author:***

In this manuscript, the authors examined the differences in volume and T1 value of the habenula (Hb) between 21 patients with major depressive disorder and 20 healthy control (HC) subjects. Structural magnetic resonance image data were obtained using a 7-T scanner. Two researchers blinded to the clinical data manually delineated the habenular nuclei and Hb volume, and T1 values were calculated based on overlapping voxels. They found the MDD patients have a smaller right Hb than HC, and that the volume of the right Hb was significantly smaller than that of the left Hb in the MDD group. Generally speaking, this is a well conducted study through my main concern for it the small sample size which may deter the robustness of the results.

**Comment #1.**

Introduction: A stronger rationale for the clinical significance of volume and T1 value is needed.

**Response**: Thank you for your comment. As per your suggestion, we have revised the manuscript accordingly. The revised Introduction now reads as follows (Introduction section, page 5, lines 78–85): “Unmedicated patients with bipolar depression showed significantly smaller Hb volumes than an HC group, and currently depressed females with MDD had smaller Hb volumes than healthy females [22]. In another study, hemispheric Hb volumes did not differ between medicated and unmedicated MDD patients and HCs; however, there were significant positive correlations between Hb volumes and depression severity [21]. A recent analysis showed that women with first-episode MDD had higher overall Hb white matter volumes than did healthy controls and patients with treatment-resistant/chronic MDD [20].”

Additionally, (Introduction section, page 6, line 95 – page 7, line 115): “In addition, a new research topic with clinical significance besides asymmetry in the Hb is myelin and T1 relaxation time, which is a measure of how quickly the net magnetization vector recovers to its ground state. After the radiofrequency (RF) pulse is stopped, the absorbed energy is released to the surrounding tissue, and the protons are rearranged in the direction of the external magnetic field in equilibrium [26, 27]. T1 relaxation time is known to be sensitive to the water content in tissues and the macromolecular tissue environment. Changes in water composition can lead to obvious changes in the T1 relaxation time, and measurement of the T1 relaxation time provides a quantitative indicator of the physiological state of the underlying tissue [28, 29]. These T1 relaxation constants have been widely used to study high-intensity lesions such as multiple sclerosis and are used as markers of tissue characteristics in MR imaging studies [30]. A previous study using the T1 relaxation time was also conducted in patients with depression, and unipolar depression patients showed a decreased T1 in the hippocampus compared to healthy controls [31]. In that study, the researchers suggested that the biophysical tissue change might be due to depression [31]. In addition, quantitative T1 mapping has shown the possibility of elucidating cortical myelin content [32], especially in high-resolution 7-T MRI [33]. Strotmann et al. concluded that the low T1 value of the Hb was due to the unusually large number of myelinated fibers [34]. It is likely that decreases in R1 (R1 = 1 / T1 value) are attributable to decreases in myelin in depression [35]. In this context, investigators have begun to use T1 values and R1 as an in vivo assay of myelin content in the Hb [35, 36].”

**Comment #2.**

Methods: Please provide more information about the MDD patients, such as illness duration.

**Response**: As per your suggestion, we have described the clinical information of patients with MDD in the revised manuscript and Table 1 as follows (page 14, line 259 - page 15, line 277): “In addition, the BHS, CGI, and SSI scores were also higher in patients with MDD than in HCs (*P* < 0.001, see **Table 1**). Compared to the control group, the depressed group also reported more hopelessness and suicidal ideation, and the overall clinical impression was more severe. When the severity of the MDD patients was classified according to the DSM-5, the number of subjects classified as severe, moderate, and mild severity in the MDD group was 6 (19%), 18 (54%), and 9 (27%), respectively. In the MDD group, the average duration of the current episode was 73.93 weeks, and the average disease duration was 63.45 months. Eighty-two percent of the MDD group were taking antidepressants, and the average duration of antidepressant use was 82.18 weeks (**Table 1**).”

**Table 1. Demographic and clinical characteristics of the MDD and HC groups**

| Clinical variables | MDD (n = 33) | HC (n = 36) | *P* values |
| --- | --- | --- | --- |
| Age at scan, years (mean ± SD) | 40.58 ± 14.16 | 35.17 ± 12.00 | 0.093^a^ |
| Sex (male:female) | 8:25 | 12:24 | 0.406^b^ |
| Education, years (mean ± SD) | 12.73 ± 3.45 | 15.22 ± 1.91 | 0.001^a^ |
| Severity specifiers in DSM-5, N (%) |  |  |  |
| Mild | 9 (27) | N/A | N/A |
| Moderate | 18 (54) | N/A | N/A |
| Severe | 6 (19) | N/A | N/A |
| Duration of current depressive episode, weeks (mean ± SD) ^¶^ | 73.93 ± 16.12 | N/A | N/A |
| Duration of illness, months (mean ± SD) | 63.45 ± 57.45 | N/A | N/A |
| The use of antidepressants, N (%) | 27 (82) | N/A | N/A |
| Duration of antidepressant use, weeks (mean ± SD) | 82.18 ± 26.24 | N/A | N/A |
| Recurrence, N (%) | 20 (60) | N/A | N/A |
| Treatment resistance, N (%) | 6 (18) | N/A | N/A |
| HDRS-17 score (mean ± SD) | 15.95 ± 5.40 | 2.53 ± 2.41 | < 0.001^a^ |
| BHS score (mean ± SD) | 12.52 ± 5.93 | 1.19 ± 1.47 | < 0.001^a^ |
| BDI score (mean ± SD) | 27.79 ± 12.66 | 3.50 ± 3.68 | < 0.001^a^ |
| CGI score (mean ± SD) | 4.03 ± 0.95 | 1.03 ± 0.17 | < 0.001^a^ |
| SSI score (mean ± SD) | 14.00 ± 8.69 | 1.00 ± 1.53 | < 0.001^a^ |

^a^ Student’s t-test; ^b^ Chi-square test; ^*^ indicates significant difference (*P* < 0.05).

Data are presented as means ± standard deviations or numbers (percentages) unless otherwise indicated. ^¶^There were five missing data on the duration of the current depressive episode.

Abbreviations: BAI, Beck Anxiety Inventory; BDI, Beck depression inventory; BHS, Beck Hopelessness Scale; CGI, clinical global impression scale; DSM-5, Fifth Edition of the Diagnostic and Statistical Manual of Mental Disorders; HDRS, Hamilton Depression Rating Scale; HC, Healthy Control; MDD, Major Depressive Disorder; SD, Standard Deviation; SSI, Scale for Suicide Ideation.

**Comment #3.**

Methods: How to guarantee the accuracy of manually delineation of the habenula?

**Response**: As mentioned in the manuscript, two examiners manually drew the borders of the habenula and their overlapping ratio of the manually segmented whole Hb was calculated to ensure that the overlap was more than 70%. Then, as suggested by a previous study [44], the area was confined to the overlapped areas, so as not to overestimate the habenular volume. The manuscript now reads as follows (page 10, line 177 – page 11, line 196): “Two well-trained researchers performed the manual delineation of the right and left Hbs using the 7-T MR images (i.e., T1 map) from the subjects. At first, the left and the right Hb were identified based on the selected slices in each data. They were manually traced from the T1 map image according to the following procedure. Using MRIcron and ImageJ as analytic tools, the examiners were able to see the image in all three planes (sagittal, coronal, and axial) simultaneously and to segment manually the target voxels outlining the Hb surface where the signal intensity differs clearly from that of the adjacent brain tissues. These data were used to evaluate the reliability of the Hb definition. During the trace, extra care was taken to separate the Hb boundaries from adjacent, non-Hb brain tissues, such as white matter and cerebrospinal fluid (CSF). This was done as precisely as possible, as confirmed by other senior researchers who had the sufficient research experience in brain MRI. The reliability was determined using the overlap index ratio (%) suggested by a previous study [44]. The overlapping ratio of the manually segmented whole Hb was calculated to ensure that it was more than 70%. Then, the area was confined to the overlapped areas, so as not to overestimate the habenular volume [44].

Overlap Index Ratio (%) = (A ∩ B) / (A ∪ B) × 100
where A is the number of voxels selected by Examiner #1 and B is the number selected by Examiner #2. The number of voxels (#) within overlapping areas was counted, and then the volume was calculated with the following formula: # × voxel size, where is 0.65×0.65×0.65 (imaging resolution). MRIron (https://www.nitrc.org/projects/mricron) and MATLAB (The Mathworks, Inc.) software were used for segmentation and volume calculations, respectively.”

**Comment #4.**

Methods: Details are missing regarding how to calculate the T1 values for each subject.

**Response**: As per your suggestion, we have inserted the information regarding the estimation of T1 values for each subject in this revised manuscript. It now reads as follows (Methods section, page 12, lines 222–224): “Each voxel has a T1 value estimated from the acquired MRI images, which was introduced in previous studies [43, 45]. The final T1 value for each subject was the average of the T1 values within the overlapped area.”

**Comment #5.**

Methods: Did the authors control the quality of MRI data to minimize the head motion?

**Response**: As per your suggestion, we have added information on how we minimized head motion in this revised manuscript. It now reads as follows (Methods section, page 9, lines 152-155): “The subjects were asked not to move their heads while they were kept in a comfortable lying position to minimize movement during the scan. In addition, cushions were placed between the RF coil and the subject’s head to secure the head.”

**Comment #6.**

Methods: Did the authors test the normality for those continuous variables, such as age, Hb volume? We recommend plotting the mean and SD if you analyze with an unpaired t test, and the median and interquartile range if you use the nonparametric Mann-Whitney test.

**Response**: To increase the sample size, we performed manual segmentation of the habenula on additional participants. Therefore, the number of participants in the MDD and HC groups was 33 and 36, respectively. After normality was confirmed by the Kolmogorov-Smirnov method, the mean habenula volume of the two groups (MDD and control groups) was compared using a Student's t-test, and the difference between left and right habenula volume within each group was analyzed by a paired t-test. When not satisfying the homogeneity of variance using Levene’s test, the difference was tested using Welch’s t-test. We have revised the description of the statistical analysis in the Methods section (page 13, lines 234-243).

As per your suggestion, we have plotted the mean and SD in Figure 3. The mean and SD values are also shown in Tables 2 and 3 as follows:

**Table 2.** **Group differences in the volume and T1 value of the habenula segmented by both examiners between MDD and HC**

|  | Hemisphere | MDD (n = 33) | HC (n = 36) | t score | *P* values |
| --- | --- | --- | --- | --- | --- |
| Volume  (in mm^3^ ± SD) | Right | 16.43 ±3.24 | 18.54 ± 3.91 | −2.431 | 0.018^*^ |
|  | Left | 18.37 ± 3.39 | 18.47 ± 5.04 | −0.090 | 0.929 |
| T1  (value ± SD) | Right | 1200.96 ± 41.29 | 1207.84 ± 32.35 | −0.773 | 0.442 |
|  | Left | 1225.03 ± 34.81 | 1220.51 ± 31.90 | 0.564 | 0.575 |

^*^ indicates significant difference (*P* < 0.05).
The statistical analysis was performed using a Student’s t-test
Abbreviations: HC, healthy control; MDD, major depressive disorder; SD, standard deviation

**Table 3. Comparison of the volume and T1 value between right and left habenula segmented by both examiners between each group**

|  | Group | Right | Left | t score | *P* values |
| --- | --- | --- | --- | --- | --- |
| Volume  (in mm^3^ ± SD) | MDD (n = 33) | 16.43 ± 3.24 | 18.37 ± 3.39 | −2.722 | 0.010^*^ |
|  | HC (n = 36) | 18.54 ± 3.91 | 18.47 ± 5.04 | 0.092 | 0.928 |
| T1  (value ± SD) | MDD (n = 33) | 1200.96 ± 41.29 | 1225.03 ± 34.81 | −5.503 | < 0.001^*^ |
|  | HC (n = 36) | 1207.84 ± 32.35 | 1220.51 ± 31.90 | −2.225 | 0.033^*^ |

^*^ indicates significant difference (*P* < 0.05).
The statistical analysis was performed using a paired t-test.
Abbreviations: HC, healthy control; MDD, major depressive disorder; SD, standard deviation.

**Fig 3.** **Scatter plot with the mean and standard deviation of the volume and T1 value of the right and left habenula in each group****.**

Volumes or T1 value of the hemispheric habenula (Y axis) for the diagnostic groups (X axis). The graph plots the individual data points with a horizontal line superimposed at the mean and error bars showing plus and minus one SD of each group. We drew red lines between the combinations that differed significantly and marked them with an asterisk (*). Please note the smaller right habenula volume in the MDD group than the left habenula volume in the MDD group and those of the right and left habenula in HCs. Also, note that the T1 value in both the HC and MDD groups is lower in the right habenula than in the left. Abbreviations: Rt, right; Lt, left; HC, healthy control; MDD, major depressive disorder; SD, standard deviation.

**Comment #7.**

Methods: The authors can perform correlation analyses between clinical scale scores and Hb volume and T1 values to link the MRI data to clinical practice.

**Response**: As you advised, to investigate the correlation between the clinical scale scores and Hb volume and T1 values in the depressed group, partial correlation analysis controlling for sex and age was performed. There was no significant correlation among the clinical scale scores, including HDRS-17 score, Hb volume, and T1 values.

In addition, we performed multiple linear regression analysis to determine whether demographic and clinical characteristics affect the habenula volume of the MDD group. In multivariate regression analyses, variables such as age, sex, years of education, the use of antidepressants, duration of antidepressant use, duration of illness, recurrence of depression, HDRS-17 score, SSI score, and severity of depression were set as independent variables with the stepwise method as they were significantly correlated with whole habenula volume as dependent variable.

In the MDD group, whole Hb volume was associated with age (β =-0.371, *p* =0.011), male sex (β =-0.485, *p* =0.001), years of education (β =0.299, *p* =0.036), and duration of antidepressant use (β =0.331, *p* =0.014; **Table 4**).

We described this issue in the Materials and methods, Results, and Discussion sections and show the statistics in Table 4.

Materials and methods section (page 13, lines 244-250): “In addition, we performed multiple linear regression analysis to determine whether demographic and clinical characteristics affect the Hb volume of the MDD group. In multivariate regression analyses, variables such as age, sex, years of education, the use of antidepressants, duration of antidepressant use, duration of illness, illness recurrence, HDRS-17 score, SSI score, and depression severity were set as independent variables with the stepwise method as they were significantly correlated with whole Hb volume. Whole Hb volume was used as a dependent variable in multivariate linear regression analyses.”

Results section (page 17, line 320– page 18, line 329): “The multivariate linear regression analysis showed the final model was suitable (*F* = 12.147, *P* < 0.001) with 62.3% explanatory power (adjusted R^2^ = 0.623). In the MDD group, whole Hb volume was associated with age (*P* = 0.011), sex (*P* = 0.001), years of education (*P* = 0.036), and duration of antidepressant use (*P* = 0.014). Among these independent variables, it was found that sex (β = −0.485) had the relatively highest influence on Hb volume, followed by age (β = −0.371), duration of antidepressant use (β = 0.331), and years of education (β = 0.299). (**Table 4**).”

**Table 4. Factors associated with whole habenula volume in the MDD group**

| **Independent variable** | **Unstandardized coefficients** | | **Standardized coefficient** | ***P*** |
| --- | --- | --- | --- | --- |
|  | ***B*** | **SE** | **β** |  |
| (Constant) | 1585876.702 | 132694.373 |  | **< 0.001***** |
| Age | −4578.510 | 1650.256 | −0.371 | **0.011*** |
| Sex (female)  (Reference group: male) | −195964.120 | 48694.135 | −0.485 | **0.001**** |
| Years of education | 14299.465 | 6417.061 | 0.299 | **0.036*** |
| Duration of antidepressant use | 401.717 | 150.203 | 0.331 | **0.014*** |
|  | *F* = 12.147, *P* < 0.001, adjusted R^2^ = 0.623, DW = 2.105 | | | |
| ^*^*P* < .05, ^**^*P* < .01, ^***^*P* < .001  Final models of multivariate linear regression analyses with stepwise method of whole habenula volume.  The dependent variable was habenula volume (log-transformed); independent variables were age, sex (female), years of education, the use of antidepressants (yes/no), duration of antidepressant use, duration of illness, recurrence of depression, HDRS-17 score, SSI score, and depression severity (mild/moderate/severe).  Abbreviations: DW, Durbin-Watson; HDRS, Hamilton Depression Rating Scale; SE, standard error; SSI, Scale for Suicide Ideation | | | | |

Discussion section (page 19, lines 336-339 and page 20, line 365 – page 21, line 383)” Moreover, among the depressed group, it was found that the whole Hb volume was smaller for females and those who were older than for males and those who were younger, respectively, and that the Hb volume increased as the years of education or the duration of antidepressant use was longer.”

“As with previous studies by Savitz et al. [22], it was shown that depressed women had a smaller Hb volume. Another study reported that the Hb volume was smaller when the depression was recurrent or chronic in depressed women [20]. Compared to men in the MDD group, women are affected by female sex hormones, are more sensitive to socio-cultural stressors, and have limited methods of relieving stress [49]; therefore, they might be more vulnerable to Hb volume reduction. In addition, the possibility of sex differences in Hb volume irrespective of depression cannot be excluded [50]. Unlike previous studies that did not find a correlation of Hb volume and age [20, 21], this study showed that the older the individuals in the depressed group, the smaller the volume. The older the patients with MDD, the more likely they are to live in isolation, have less interpersonal contact, and have a higher likelihood of physical illness [51], which may lead to a smaller whole Hb volume. To our knowledge, there are no research results that clearly demonstrate a correlation between Hb volume and duration of education or antidepressant medication use. Past studies suggest that use of antidepressants might have an effect on preventing neuronal damage and cell loss that occur in patients who are depressed [52]. In this context, the longer period of antidepressant use in patients with MDD might be related to a lower decrease in Hb volume. Since pubertal hormones are released and development of higher-order cognition through enhanced plasticity of cortical circuits is present in adolescents [53], the duration of education could affect the volume of the brain. Thus, the longer the education period, the greater the Hb volume. ”

**Comment #8.**

Results: I would suggest adding an iconic image of Hb rather than only axial images to provide a more comprehensive impression of this structure for audiences.

**Response**: As per your suggestion, the additional coronal images are included in Figure 1 as follows:

**Fig 1. The habenula of an HC (left panel) and MDD patient (right panel).**

(a) axial MPRAGE, (b) axial view in ME-MP2RAGE T1 maps, and (c) coronal view in ME-MP2RAGE T1 maps. Compared to the surrounding tissues, the habenula has a brighter signal on the MPRAGE image and darker signal on the ME-MP2RAGE T1 map image. Overall, the contrast on the ME-MP2RAGE T1 map image is greater than that on the MPRAGE image. The yellow arrow points to the habenula, and the boundaries of the habenula are indicated by the yellow dotted line. Abbreviations: HC, healthy control; MDD, major depressive disorder; ME-MP2RAGE, multi-echo magnetization-prepared 2 rapid gradient echoes; MPRAGE, magnetization-prepared rapid gradient echo

**Comment #9.**

Results: I would suggest adding caps to indicate the significant group difference in Figure 3.

**Response**: As per your advice, we added red caps and marked the significant group difference combinations with an asterisk(*) in Figure 3 as follows:

**Fig 3. Scatter plot with the mean and standard deviation of the volume and T1 value of the right and left habenula in each group.**

Volumes or T1 value of the hemispheric habenula (Y axis) for the diagnostic groups (X axis). The graph plots the individual data points with a horizontal line superimposed at the mean and error bars showing plus and minus one SD of each group. We drew red lines between the combinations that differed significantly and marked them with an asterisk (*). Please note the smaller right habenula volume in the MDD group than the left habenula volume in the MDD group and those of the right and left habenula in HCs. Also, note that the T1 value in both the HC and MDD groups is lower in the right habenula than in the left. Abbreviations: Rt, right; Lt, left; HC, healthy control; MDD, major depressive disorder; SD, standard deviation.

**Comment #10.**

Discussion: The authors did not discuss how these findings would impact an individual MD patient. In other words, please specify the clinical applications/value of the results.

**Response**: Thank you for your comment. Our results, obtained using high-resolution 7-T MRI, could contribute to a better understanding of the neuroanatomical characteristics of Hb in MDD. Although the results of this study are thought to contribute to clarifying the etiology of MDD patients in the future, it is not likely that brain imaging studies can be applied to biomarker for the diagnosis, treatment response, and recurrence of MDD yet. We have described the impact of our findings in the Discussion as follows (page 23, lines 422-427): “This study showed decreased right Hb volume and left-right asymmetry of the Hb volume and T1 value in MDD. Our results, obtained using high-resolution 7-T MRI, could contribute to a better understanding of the neuroanatomical characteristics of Hb in MDD. Although this study is likely to contribute to clarifying the etiology of MDD patients in the future, the brain imaging studies may not be immediately utilized as a biomarker for the diagnosis, treatment response, and recurrence of MDD [66].”

**Comment #11.**

Discussion: More space needs to be devoted to discussing the limitations of this study. For instance, the small sample size which may deter the robustness of the results.

**Response**: Thank you for your comment. Our study included a total of 69 subjects, which could still be considered relatively small. Therefore, we described the potential limitations of our study in the Discussion as per your advice. The contents are as follows (page 23, lines 427–431): “Owing to the relatively small number of subjects in this study, it is difficult to generalize our results. Additionally, the type, amount, and duration of psychotropic medications being taken by each subject in the depression group might have affected the outcome. Further replication studies with more control over such variables and larger sample sizes are needed.”

**Responses to reviewer #2**

We thank the reviewer for the comments on our manuscript, which we believe have helped us considerably to improve it. Please see below for individual responses to the reviewer’s comments.

***Comments to the Author:***

This is a paper testing hebanula volume in MDD and laterality of changes in both patients and controls. The paper focuses on n important brain structure, has a clear hypothesis and is well written.

**Comment #1.**

Introduction:

Lateral and medial hebanula is mentioned. I imagine that the size of the hebanula is too small to allow measurements of these parts separately?

**Response**: We agree with you. The previous postmortem study divided the habenula into lateral and medial parts; however, it was impossible to distinguish lateral and medial parts using the current 7 Tesla brain imaging technology. Previously, no MRI studies divided the habenula into medial and lateral parts, and the description of lateral and medial habenula in our manuscript only applies to the postmortem study.

**Comment #2.**

I am not sure if it is right to call hebanula a 'key region', we don't know yet well how crucial it is for MDD development. Perhaps 'important' would do it justice?

**Response:** As suggested, we changed “key” to “important” as follows (page 3, line 29 and page 5, line 70): “The habenula (Hb) has been hypothesized to play an essential role in major depressive disorder (MDD) as it is considered to be an important node between fronto-limbic areas and midbrain monoaminergic structures based on animal studies.”

“However, the human Hb, which is estimated to be an important brain region involved in depression, is 5–9 mm in size and neuroimaging studies are lacking because the visualization and exact delineation of this structure is not easy using conventional 3-T brain magnetic resonance imaging (MRI).”

**Comment #3.**

One of the exclusion criteria is 'no unstable or major medical or neurological disorders within the past 1 year'. Does it mean that if these were present before, e.g. someone had a stroke, they would be included?

**Response**: Thank you for this question. Those who had a previous cerebrovascular attack were excluded. To describe this more accurately, we have added the following criteria in the exclusion of the Materials and methods section as follows (page 8, line 134): “(iv) no history of cerebrovascular accident”

**Comment #4.**

Methods: Why were two different kinds of a structural image acquired?

**Response**: MPRAGE images have been the most commonly used images to differentiate the anatomical structures of the brain. However, it was difficult to distinguish small and low-contrast structures such as the habenular using MRPAGE. Therefore, images were acquired using MP2RAGE, a new imaging technique that has the advantage of obtaining T1 maps because multiple images can be acquired simultaneously. However, MPRAGE images are still needed to compare the superiority of MR2RAGE. The revised text now reads as follows (Methods section, page 9, lines 157-162): “Although magnetization-prepared rapid gradient echo (MRPAGE) is commonly used for anatomical imaging, MP2RAGE, a new imaging technique that has the advantage of obtaining T1 relaxation time maps, was used. However, it was not expected to distinguish small and low-contrast structures such as the Hb. Therefore, it was necessary to acquire both images using MPRAGE and MP2RAGE imaging techniques for comparison.”

**Comment #5.**

A description of a protocol for volume calculation is completely missing and needs to be added (which software was used, which steps were undertaken).

**Response**: We inserted information regarding the volume calculation in the revised manuscript (page 11, lines 193-196): “The number of voxels (#) within overlapping areas was counted, and then the volume was calculated with the following formula: # × voxel size, where is 0.65×0.65×0.65 (imaging resolution). MRIron (https://www.nitrc.org/projects/mricron) and MATLAB (The Mathworks, Inc.) software were used for segmentation and volume calculations, respectively.”

**Comment #6.**

Results:

The MDD group needs to be better characterised. It is important to include the time since MDD onset, possibly the number of episodes, length of the current episode, whether any patient was treatment-resistent. It would be interesting to perform exploratory correlations on the length of disorder and hebanula volume.

**Response**: As per your recommendation, we have described the characteristics of the participants in more detail in the revised manuscript as follows (page 14, lines 259-267, and Table 1): “In addition, the BHS, CGI, and SSI scores were also higher in patients with MDD than in HCs (*P* < 0.001, see **Table 1**). Compared to the control group, the depressed group also reported more hopelessness and suicidal ideation, and the overall clinical impression was more severe. When the severity of the MDD patients was classified according to the DSM-5, the number of subjects classified as severe, moderate, and mild severity in the MDD group was 6 (19%), 18 (54%), and 9 (27%), respectively. In the MDD group, the average duration of the current episode was 73.93 weeks, and the average disease duration was 63.45 months. Eighty-two percent of the MDD group were taking antidepressants, and the average duration of antidepressant use was 82.18 weeks (**Table 1**).”

**Table 1. Demographic and clinical characteristics of the MDD and HC groups**

| Clinical variables | MDD (n = 33) | HC (n = 36) | *P* values |
| --- | --- | --- | --- |
| Age at scan, years (mean ± SD) | 40.58 ± 14.16 | 35.17 ± 12.00 | 0.093^a^ |
| Sex (male:female) | 8:25 | 12:24 | 0.406^b^ |
| Education, years (mean ± SD) | 12.73 ± 3.45 | 15.22 ± 1.91 | 0.001^a^ |
| Severity specifiers in DSM-5, N (%) |  |  |  |
| Mild | 9 (27) | N/A | N/A |
| Moderate | 18 (54) | N/A | N/A |
| Severe | 6 (19) | N/A | N/A |
| Duration of current depressive episode, weeks (mean ± SD) ^¶^ | 73.93 ± 16.12 | N/A | N/A |
| Duration of illness, months (mean ± SD) | 63.45 ± 57.45 | N/A | N/A |
| The use of antidepressants, N (%) | 27 (82) | N/A | N/A |
| Duration of antidepressant use, weeks (mean ± SD) | 82.18 ± 26.24 | N/A | N/A |
| Recurrence, N (%) | 20 (60) | N/A | N/A |
| Treatment resistance, N (%) | 6 (18) | N/A | N/A |
| HDRS-17 score (mean ± SD) | 15.95 ± 5.40 | 2.53 ± 2.41 | < 0.001^a^ |
| BHS score (mean ± SD) | 12.52 ± 5.93 | 1.19 ± 1.47 | < 0.001^a^ |
| BDI score (mean ± SD) | 27.79 ± 12.66 | 3.50 ± 3.68 | < 0.001^a^ |
| CGI score (mean ± SD) | 4.03 ± 0.95 | 1.03 ± 0.17 | < 0.001^a^ |
| SSI score (mean ± SD) | 14.00 ± 8.69 | 1.00 ± 1.53 | < 0.001^a^ |

^a^ Student’s t-test; ^b^ Chi-square test; ^*^ indicates significant difference (*P* < 0.05).

Data are presented as means ± standard deviations or numbers (percentages) unless otherwise indicated. ^¶^There were five missing data on the duration of the current depressive episode.

Abbreviations: BAI, Beck Anxiety Inventory; BDI, Beck depression inventory; BHS, Beck Hopelessness Scale; CGI, clinical global impression scale; DSM-5, Fifth Edition of the Diagnostic and Statistical Manual of Mental Disorders; HDRS, Hamilton Depression Rating Scale; HC, Healthy Control; MDD, Major Depressive Disorder; SD, Standard Deviation; SSI, Scale for Suicide Ideation.

In addition, as you advised, an exploratory partial correlation analysis controlling for sex and age was conducted between clinical characteristics and Hb volume and T1 values in the depressed group. There was no signiﬁcant correlation between the duration of the current episode and Hb volume.

**Comment #7.**

It is very important to know whether patients were treated with medications and if so, which medications, as medications may have an impact on both function and structure of the brain regions. The impact of medications should be noted.

**Response**: As you pointed out, there is a possibility that the medication could affect the brain volume and function. In the MDD group, 27 participants were taking antidepressants. The effects of clinical information, including medication usage, on the habenula volume were assessed using multiple stepwise regression analysis after performing correlation analysis. A p-value of less than 0.05 (*p* < 0.05) was considered significant.

We described this issue in the Materials and methods, Results, and Discussion sections and show the statistics in Table 4.

Materials and methods section (page 13, lines 244-250): “In addition, we performed multiple linear regression analysis to determine whether demographic and clinical characteristics affect the Hb volume of the MDD group. In multivariate regression analyses, variables such as age, sex, years of education, the use of antidepressants, duration of antidepressant use, duration of illness, illness recurrence, HDRS-17 score, SSI score, and depression severity were set as independent variables with the stepwise method as they were significantly correlated with whole Hb volume. Whole Hb volume was used as a dependent variable in multivariate linear regression analyses.”

Results section (page 17, line 320 – page 18, line 329): “The multivariate linear regression analysis showed the final model was suitable (*F* = 12.147, *P* < 0.001) with 62.3% explanatory power (adjusted R^2^ = 0.623). In the MDD group, whole Hb volume was associated with age (*P* = 0.011), sex (*P* = 0.001), years of education (*P* = 0.036), and duration of antidepressant use (*P* = 0.014). Among these independent variables, it was found that sex (β = −0.485) had the relatively highest influence on Hb volume, followed by age (β = −0.371), duration of antidepressant use (β = 0.331), and years of education (β = 0.299). (**Table 4**). “

**Table 4. Factors associated with whole habenula volume in the MDD group**

| **Independent variable** | **Unstandardized coefficients** | | **Standardized coefficient** | ***P*** |
| --- | --- | --- | --- | --- |
|  | ***B*** | **SE** | **β** |  |
| (Constant) | 1585876.702 | 132694.373 |  | **< 0.001***** |
| Age | −4578.510 | 1650.256 | −0.371 | **0.011*** |
| Sex (female)  (Reference group: male) | −195964.120 | 48694.135 | −0.485 | **0.001**** |
| Years of education | 14299.465 | 6417.061 | 0.299 | **0.036*** |
| Duration of antidepressant use | 401.717 | 150.203 | 0.331 | **0.014*** |
|  | *F* = 12.147, *P* < 0.001, adjusted R^2^ = 0.623, DW = 2.105 | | | |
| ^*^*P* < .05, ^**^*P* < .01, ^***^*P* < .001  Final models of multivariate linear regression analyses with stepwise method of whole habenula volume.  The dependent variable was habenula volume (log-transformed); independent variables were age, sex (female), years of education, the use of antidepressants (yes/no), duration of antidepressant use, duration of illness, recurrence of depression, HDRS-17 score, SSI score, and depression severity (mild/moderate/severe).  Abbreviations: DW, Durbin-Watson; HDRS, Hamilton Depression Rating Scale; SE, standard error; SSI, Scale for Suicide Ideation | | | | |

Discussion section (page 19, lines 336-339 and page 20, line 365 – page 21, line 383) “Moreover, among the depressed group, it was found that the whole Hb volume was smaller for females and those who were older than for males and those who were younger, respectively, and that the Hb volume increased as the years of education or the duration of antidepressant use was longer.”

“As with previous studies by Savitz et al. [22], it was shown that depressed women had a smaller Hb volume. Another study reported that the Hb volume was smaller when the depression was recurrent or chronic in depressed women [20]. Compared to men in the MDD group, women are affected by female sex hormones, are more sensitive to socio-cultural stressors, and have limited methods of relieving stress [49]; therefore, they might be more vulnerable to Hb volume reduction. In addition, the possibility of sex differences in Hb volume irrespective of depression cannot be excluded [50]. Unlike previous studies that did not find a correlation of Hb volume and age [20, 21], this study showed that the older the individuals in the depressed group, the smaller the volume. The older the patients with MDD, the more likely they are to live in isolation, have less interpersonal contact, and have a higher likelihood of physical illness [51], which may lead to a smaller whole Hb volume. To our knowledge, there are no research results that clearly demonstrate a correlation between Hb volume and duration of education or antidepressant medication use. Past studies suggest that use of antidepressants might have an effect on preventing neuronal damage and cell loss that occur in patients who are depressed [52]. In this context, the longer period of antidepressant use in patients with MDD might be related to a lower decrease in Hb volume. Since pubertal hormones are released and development of higher-order cognition through enhanced plasticity of cortical circuits is present in adolescents [53], the duration of education could affect the volume of the brain. Thus, the longer the education period, the greater the Hb volume.”

**Comment #8.**

In my opinion, the fragment starting at page 11, line 12 ('In the data obtained from...') up to page 13, line 3 belongs to Methods rather that results.

**Response**: We agree with your comment. We moved the parts from the Results to the Methods section (page 11, line 197– page 12, line 231) as per your advice. Thank you for your comment.

**Comment #9.**

The Authors mention results for T1 but for an average reader it may be unclear what T1 means and why, of all the values, this one was presented as relevant to the subject. A sentence of explanation is needed.

**Response**: As per your suggestion, we added an explanation for the T1. It now reads as follows (page 6, line 95 – page 7, line 115): “In addition, a new research topic with clinical significance besides asymmetry in the Hb is myelin and T1 relaxation time, which is a measure of how quickly the net magnetization vector recovers to its ground state. After the radiofrequency (RF) pulse is stopped, the absorbed energy is released to the surrounding tissue, and the protons are rearranged in the direction of the external magnetic field in equilibrium [26, 27]. T1 relaxation time is known to be sensitive to the water content in tissues and the macromolecular tissue environment. Changes in water composition can lead to obvious changes in the T1 relaxation time, and measurement of the T1 relaxation time provides a quantitative indicator of the physiological state of the underlying tissue [28, 29]. These T1 relaxation constants have been widely used to study high-intensity lesions such as multiple sclerosis and are used as markers of tissue characteristics in MR imaging studies [30]. A previous study using the T1 relaxation time was also conducted in patients with depression, and unipolar depression patients showed a decreased T1 in the hippocampus compared to healthy controls [31]. In that study, the researchers suggested that the biophysical tissue change might be due to depression [31]. In addition, quantitative T1 mapping has shown the possibility of elucidating cortical myelin content [32], especially in high-resolution 7-T MRI [33]. Strotmann et al. concluded that the low T1 value of the Hb was due to the unusually large number of myelinated fibers [34]. It is likely that decreases in R1 (R1 = 1 / T1 value) are attributable to decreases in myelin in depression [35]. In this context, investigators have begun to use T1 values and R1 as an in vivo assay of myelin content in the Hb [35, 36].”

**Comment #10.**

p.13 line 6 - the (mean?) overlapping volumes.

**Response**: Yes, it is the mean volume. We have edited this accordingly (page 15, line 280).

**Comment #11.**

It is worth mentioning that there are scatterplots showing results for individual patients and healthy controls.

**Response**: As per your suggestion, we have changed Figure 3 to the scatter plot, including the mean and standard deviation, and we drew red lines between the combinations that differed significantly and marked them with an asterisk(*) as follows:

**Fig 3. Scatter plot with the mean and standard deviation of the volume and T1 value of the right and left habenula in each group.**

Volumes or T1 value of the hemispheric habenula (Y axis) for the diagnostic groups (X axis). The graph plots the individual data points with a horizontal line superimposed at the mean and error bars showing plus and minus one SD of each group. We drew red lines between the combinations that differed significantly and marked them with an asterisk (*). Please note the smaller right habenula volume in the MDD group than the left habenula volume in the MDD group and those of the right and left habenula in HCs. Also, note that the T1 value in both the HC and MDD groups is lower in the right habenula than in the left. Abbreviations: Rt, right; Lt, left; HC, healthy control; MDD, major depressive disorder; SD, standard deviation.

As suggested, we have added an explanation of the scatter plot in the Results section as follows (page 15, line 285 – page 16, line 286): “In **Figure 3**, the volume and T1 values of the right and left Hb in each group are visualized as scatter plots including the mean and SD.”

**Comment #12.**

Discussion

p. 17 line 18 The sentence starting with 'Laterality difference...' is not clear. Does it mean that laterality is necessary to preserve good somatosensory processing in MDD? Or the opposite?

**Response**: We apologize for the confusion. As per your advice, we have modified the sentences to convey the meaning more clearly as follows (page 21, line 398 – page 22, line 401): “Laterality differences might be necessary to preserve efficient somatosensory processing in MDD [58] and various neurological conditions are associated with loss in the lateralization of brain activity [59-61]; however, it is unclear whether these abnormalities are a cause or consequence of the disease.”

**Comment #13.**

The Authors mention advantages of this study but not limitations. Could you please add a paragraph critically assessing limitations of the study?

**Response**: Thank you for your comment. As per your advice, we have described the limitations of our study in the Discussion section as follows (page 23, lines 424-431): “Although this study is likely to contribute to clarifying the etiology of MDD patients in the future, the brain imaging studies may not be immediately utilized as a biomarker for the diagnosis, treatment response, and recurrence of MDD [66]. Owing to the relatively small number of subjects in this study, it is difficult to generalize our results. Additionally, the type, amount, and duration of psychotropic medications being taken by each subject in the depression group might have affected the outcome. Further replication studies with more control over such variables and larger sample sizes are needed.”

***Reference***

44.	J. Pantel DSOL, K. Cretsinger, H. J. Bockholt, H. Keefe, V. A. Magnotta, and N. C. Andreasen. A new method for the in vivo volumetric measurement of the human hippocampus with high neuroanatomical accuracy. Hippocampus. 2000;10(6).
