# Supplementary material for: Left-right asymmetric and smaller right habenula volume in major depressive disorder on high-resolution 7-T magnetic resonance imaging
Source: PLoS One. 2021 Aug 3;16(8):e0255459. doi: 10.1371/journal.pone.0255459 (PMC8330903; doi:10.1371/journal.pone.0255459)
Supplement: Attachment — Submitted filename: Response to reviewers_R2.docx [file pone.0255459.s003.docx]

**Responses to Reviewer #2:**
We deeply appreciate the reviewer’s positive comments on our article.

**Review Comments to the Autho*r:***

The Authors have addressed my comments in an adequate manner. The manuscript reads well and the methodology and results are presented in a sound way.

The introduction and discussion have benefitted from information added.

I can recommend the manuscript for publication.

**Responses to Reviewer #3:**

We thank the reviewer for their comments regarding our article. We believe that this article has been considerably improved based on the reviewer’s comments and suggestions. Please see below for individual responses to their comments:

**Review Comments to the Autho*r:***

Please, find a few comments below for your perusal:

**Comment #1.**

Introduction: It would be best if you added a reference to the first sentence. It would seem that you are referring to the DSM 5, so please, add the citation. This is not necessarily a universal definition.

**Response**: We agree with your comment. As per your advice, we added the DSM-5 reference to the first sentence of the Introduction section (page 4, line 47; page 28, line 490).

**Comment #2.**

Methods: Please, specify if there was a measure of inter-rater reliability among assessors and if not known, please, add it to the limitations list.

**Response**: The volumes of the segmented structures were utilized for inter-rater reliability (interclass correlation), and the results have been added to the revised manuscript.

|  | Right | p-value | Left | p-value |
| --- | --- | --- | --- | --- |
| ICC (r) | 0.703 | p<0.001 | 0.656 | p<0.001 |

r indicates the correlation coefficients.

We have described this issue in the Materials and methods (page 11, lines 203–204) and Results (page 17, lines 314–315) sections.

Material and methods section (page 11, line 203-206): “The volumes of the segmented structures were utilized for inter-rater reliability (interclass correlation [ICC]), and the overlapping ratio of the manually segmented whole Hb was calculated to ensure that it was more than 70%.”

Results section (page 17, line 314–315): “The ICC coefficients were 0.703 (*P* < 0.001) and 0.656 (*P* < 0.001) in the right and left Hb, respectively.”

**Comment #3.**

Please, specify if an instrument was used for the diagnostic interview e.g. SCID or MINI and how comorbidity including personality disorders was excluded.

**Response**: Thank you for your comment. As mentioned in the manuscript, board-certified psychiatrists interviewed the participants to assess their eligibility for the study using the Structured Clinical Interview for Diagnostic and Statistical Manual of Mental Disorders, 5^th^ edition (DSM-5) (SCID). In addition, after the participants completed a self-report patient questionnaire for the SCID-5-personality disorder (SCID-5-SPQ) [39], the clinicians reviewed it and asked additional questions to exclude any participants with personality disorder. This information is now included in the Material and methods section (page 8, lines 130–133).

**Comment #4.**

How was mental illness excluded in healthy controls? Did you use a standardized instrument? If nit this is a limitation to be added to the list.

**Response**: As in the above answer, we used the SCID for all participants, and only those who were confirmed not to have mental illness were included in the healthy control group.

**Comment #5.**

Please, mention all the variables which were extracted from patients and controls irrespective if these were matched or not.

**Response**: As per your suggestion, we have described all the variables that were extracted from patients and controls as follows (Methods section, pages 13–14, lines 251–256): “Demographic data and clinical characteristics, including age, sex, education level, and scores of the clinician-administered scales of the HDRS-17, BHS, BDI, CGI, and SSI were summarized and compared using a Student’s t-test or chi-square test. In the MDD group, the number of depressive episodes, duration of current depressive episode, duration of illness, whether taking antidepressants, the duration of antidepressant use, and the subgroups (first episode, treatment-resistance, recurrence, and remission) of MDD were described.”

We also show this clinical information and a comparison between the groups in Table 1.

**Table 1. Demographic and clinical characteristics of the MDD and HC groups**

| Clinical variables | MDD (n = 33) | HC (n = 36) | *P* values |
| --- | --- | --- | --- |
| Age at scan, years (mean ± SD) | 40.58 ± 14.16 | 35.17 ± 12.00 | 0.093^a^ |
| Sex (male : female) | 8 : 25 | 12 : 24 | 0.406^b^ |
| Education, years (mean ± SD) | 12.73 ± 3.45 | 15.22 ± 1.91 | 0.001^a^ |
| Severity ranges for the HDRS-17 score, n (%) |  |  |  |
| Within the normal range (0–6) | 2 (6) | 36 (100) | N/A |
| Mild depression (7­–17) | 14 (42) | 0 | N/A |
| Moderate depression (18–24) | 13 (39) | 0 | N/A |
| Severe depression (≥ 25) | 4 (12) | 0 | N/A |
| The number of episodes (mean ± SD) | 2.58 ± 1.39 | N/A | N/A |
| Duration of current depressive episode, weeks (mean ± SD) | 73.93 ± 16.12 | N/A | N/A |
| Duration of illness, months (mean ± SD) | 63.45 ± 57.45 | N/A | N/A |
| The use of antidepressants, n (%) | 27 (82) | N/A | N/A |
| Duration of antidepressant use, weeks (mean ± SD) | 82.18 ± 26.24 | N/A | N/A |
| MDD subgroups^c^ |  |  |  |
| First episode, n (%) | 12 (36) | N/A | N/A |
| Treatment-resistance, n (%) | 6 (18) | N/A | N/A |
| Recurrence, n (%) | 15 (45) | N/A | N/A |
| Remission, n (%) | 2 (6) | N/A | N/A |
| HDRS-17 score (mean ± SD) | 15.95 ± 5.40 | 2.53 ± 2.41 | < 0.001^a^ |
| BHS score (mean ± SD) | 12.52 ± 5.93 | 1.19 ± 1.47 | < 0.001^a^ |
| BDI score (mean ± SD) | 27.79 ± 12.66 | 3.50 ± 3.68 | < 0.001^a^ |
| CGI score (mean ± SD) | 4.03 ± 0.95 | 1.03 ± 0.17 | < 0.001^a^ |
| SSI score (mean ± SD) | 14.00 ± 8.69 | 1.00 ± 1.53 | < 0.001^a^ |

^a^ Student’s t-test; ^b^ Chi-square test; ^c^ Some patients belonged to more than one group.

Data are presented as means ± standard deviations or numbers (percentages) unless otherwise indicated.

Abbreviations: BAI, Beck Anxiety Inventory; BDI, Beck depression inventory; BHS, Beck Hopelessness Scale; CGI, clinical global impression scale; HDRS, Hamilton Depression Rating Scale; HC, Healthy Control; MDD, Major Depressive Disorder; SD, Standard Deviation; SSI, Scale for Suicide Ideation.

**Comment #6.**

Were the patients experiencing unipolar or bipolar depression?

**Response**: We included only patients with unipolar depression in the MDD group. Patients who had hypomanic or manic episodes were excluded. We described this Method section (page 9, line 144).

**Comment #7.**

We would need to know more about the number of episodes and whether you included treatment resistant patients.

**Response**: As suggested, we described the number of depressive episodes and the proportion of treatment-resistant patients as follows (Results section, page 16, lines 291–294, Table 1): “In the MDD group, the average duration of the current episode was 73.93 weeks, the average number of depressive episodes was 2.58 (SD 1.39), the proportion of treatment-resistant patients was 15%, and the average disease duration was 63.45 months.”

We also show this clinical information and a comparison between the groups (Table 1).

**Comment #8.**

Which level of depression did you include? Please, provide the cut off for inclusion according to the rating scale you used. The DSM 5 does not provide a continuous measure of severity. You will need to use the rating scale score for that. This is pretty customary and should be established before inclusion at the stage of study design.

**Response**: We included participants who met the DSM-5 criteria for MDD throughout their lifetime in the MDD group, regardless of the severity of the current episode. We rated the severity of their current depression based on the score from the Korean version of the HDRS-17 [41] as follows: within the normal range (0–6), mild depression (7–17), moderate depression (18–24), and severe depression (≥ 25). We also divided the participants into four subgroups according to subgroup criteria described in the manuscript and quoted below.

As per your suggestion, we have described the depression rating scale, severity scoring criteria, and number of participants in each subgroup in the revised manuscript as follows:

Methods section, page 9, line 143: “The MDD patients who met the diagnostic criteria for MDD as stated in the DSM-5 [40] were included. Patients who had hypomanic or manic episodes were excluded. The MDD group was divided into four subgroups as follows: (i) first episode (those who have experienced the first episode of MDD and have not previously taken psychotropic drugs such as antidepressants, and have a 17-item Hamilton Depression Rating Scale (HDRS-17) score of 7 or higher); (ii) treatment-resistance (those who have experienced major depressive episodes for more than two years in their lifetime and who have sustained major depressive episodes that do not show treatment response to two or more antidepressants); (iii) recurrence (those with two or more episodes of major depressive episodes and have not taken psychiatric medications such as antidepressants for more than one month); and (iv) remission (those with an HDRS-17 score ≤ 6 over the last 2 months or more).”

Methods section, page 9, line 161: “As per a previous study [41], severity on the Korean version of the HDRS-17 was defined based on the total score as follows: within the normal range (0–6), mild depression (7–17), moderate depression (18–24), and severe depression (≥ 25).”

Result section, page 16, line 294: “The MDD group was divided into four subgroups: first episode (n = 12), treatment-resistance (n = 6), recurrence (n = 15), and remission (n = 2). Since some patients belonged to more than one group, the sum of the number of participants of each group exceeded the total number of patients.”

**Comment #9.**

Please, mention which medications patients were taking or if they underwent any other treatment. You would need to specify the time frame between initiation of treatment and time of scanning because medication has most likely an effect on brain structure. Were patients stable on medication and if yes for how long? The above could be added to the discussion as a limitation. See for example:

Arnone, D., McKie, S., Elliott, R. et al. State-dependent changes in hippocampal grey matter in depression. Mol Psychiatry 18, 1265–1272 (2013). https://doi.org/10.1038/mp.2012.150

**Response**: As per your suggestion, we have described the antidepressants that the patients had been taking and the period of time between initiation of antidepressant therapy and time of scanning.

It now reads as follows (page 16, lines 297–301): “Eighty-two percent of the MDD group were taking antidepressants, and the average duration of antidepressant use was 82.18 weeks (**Table 1**). The main antidepressants in patients with MDD were escitalopram (n = 12), vortioxetine (n = 3), bupropion (n = 2), desvenlafaxine (n = 2), fluoxetine (n = 2), sertraline (n = 2), milnacipran (n = 2), and mirtazapine (n = 2).”

As you pointed out, there is a possibility that medication could affect brain function and volume. Therefore, the effects of clinical characteristics, including medication usage, on the habenula volume were assessed using multiple stepwise regression analysis after performing correlation analysis. A *P* value < 0.05 was considered significant. We described this issue in the Materials and methods, Results, and Discussion sections and show the statistics in Table 4. We also cited the article (reference #55, Arnone et al., 2013) you suggested in the Discussion section as supporting evidence that antidepressants might affect brain structure.

Materials and methods section (page 14, lines 269–275): “In addition, we performed multiple linear regression analysis to determine whether demographic and clinical characteristics affect the Hb volume of the MDD group. In multivariate regression analyses, variables such as age, sex, years of education, the use of antidepressants, duration of antidepressant use, duration of illness, illness recurrence, HDRS-17 score, SSI score, and depression severity were set as independent variables with the stepwise method as they were significantly correlated with whole Hb volume. Whole Hb volume was used as a dependent variable in multivariate linear regression analyses.”

Results section (page 20, line 361–367): “The multivariate linear regression analysis showed the final model was suitable (*F* = 12.147, *P* < 0.001) with 62.3% explanatory power (adjusted R^2^ = 0.623). In the MDD group, whole Hb volume was associated with age (*P* = 0.011), sex (*P* = 0.001), years of education (*P* = 0.036), and duration of antidepressant use (*P* = 0.014). Among these independent variables, it was found that sex (β = −0.485) had the relatively highest influence on Hb volume, followed by age (β = −0.371), duration of antidepressant use (β = 0.331), and years of education (β = 0.299). (**Table 4**).”

**Table 4. Factors associated with whole habenula volume in the MDD group**

| **Independent variable** | **Unstandardized coefficients** | | **Standardized coefficient** | ***P*** |
| --- | --- | --- | --- | --- |
|  | ***B*** | **SE** | **β** |  |
| (Constant) | 1585876.702 | 132694.373 |  | **< 0.001***** |
| Age | −4578.510 | 1650.256 | −0.371 | **0.011*** |
| Sex (female)  (Reference group: male) | −195964.120 | 48694.135 | −0.485 | **0.001**** |
| Years of education | 14299.465 | 6417.061 | 0.299 | **0.036*** |
| Duration of antidepressant use | 401.717 | 150.203 | 0.331 | **0.014*** |
|  | *F* = 12.147, *P* < 0.001, adjusted R^2^ = 0.623, DW = 2.105 | | | |
| ^*^*P* < .05, ^**^*P* < .01, ^***^*P* < .001  Final models of multivariate linear regression analyses with stepwise method of whole habenula volume.  The dependent variable was habenula volume (log-transformed); independent variables were age, sex (female), years of education, the use of antidepressants (yes/no), duration of antidepressant use, duration of illness, recurrence of depression, HDRS-17 score, SSI score, and depression severity (mild/moderate/severe).  Abbreviations: DW, Durbin-Watson; HDRS, Hamilton Depression Rating Scale; SE, standard error; SSI, Scale for Suicide Ideation | | | | |

Discussion section (page 22, lines 380): “Moreover, among the depressed group, it was found that the whole Hb volume was smaller for females and those who were older than for males and those who were younger, respectively, and that the Hb volume increased as the years of education or the duration of antidepressant use was longer.”

Discussion section (page 24, lines 419–427): “To our knowledge, there are no research results that clearly demonstrate a correlation between Hb volume and duration of education or antidepressant medication use. Past studies suggest that use of antidepressants might have an effect on preventing neuronal damage and cell loss that occur in patients who are depressed [54, 55]. In this context, the longer period of antidepressant use in patients with MDD might be related to a lower decrease in Hb volume. Since pubertal hormones are released and development of higher-order cognition through enhanced plasticity of cortical circuits is present in adolescents [56], the duration of education could affect the volume of the brain. Thus, the longer the education period, the greater the Hb volume.”

We also described this issue as a limitation as follows (page 26, lines 483–484): “Additionally, the type, amount, and duration of psychotropic medications being taken by each subject in the depression group might have affected the outcome.”

**Comment #10.**

Please, report the inter-rater reliability for the operators who measured the habenula to show that their measurements were concordant.

**Response**: As we responded to Comment #2, the volumes of the segmented structures were utilized for inter-rater reliability (interclass correlation), and the results have been added to the revised manuscript.

|  | Right | *P*-value | Left | *P*-value |
| --- | --- | --- | --- | --- |
| ICC (r) | 0.703 | *P* < 0.001 | 0.656 | *P* < 0.001 |

r indicates the correlation coefficients.

We have described this issue in the Materials and methods (page 11, lines 203–204) and Results (page 17, lines 314–315) sections.

**Comment #11.**

Statistical analysis: The approach is unusual. The obvious methodology would be to run an ANCOVA or MANOVA, include left and right structures, healthy controls and depressed subjects in one analysis and control for the confounders within the analysis and not subsequently to check the effect of confounders with a regression model. The regression indicates that confounders had an effect by the way which is concerning in relation to the validity of your t-tests. You would also need to take into account the multiple comparisons (t-tests don’t do that) and the effects of brain volume sex and age (even if matched). Where the patient all right or left handed? If not this is another confounder. In this study particularly, patients had lower level of education vs. controls and were not matched for IQ, it may well be that whole brain morphometry has an impact. Please, run the analysis controlling for potential confounders within the comparisons of the habenula. The analysis you run is not sufficiently valid or strong to support your conclusions. This is consistent with our initial study results, where the difference in the volume of the right habenula significantly differed between the two groups.

**Response**: As described in the paper, all subjects were right-handed, since the common eligibility criteria included ‘right-handed using the Edinburgh Handedness Test’ (page 8, lines 135–136).

As per your suggestion, we performed an ANCOVA to compare habenula volume while controlling for potential confounding factors (i.e., age, sex, education level, and total intracranial volume). Since individual differences in whole-brain volume could have been a confounder in the analysis, we divided each habenula volume by the whole brain volume and analyzed the volume ratio. The normalization of the Hb volumes was performed using the total intracranial volume (ICV) from the 3T MRI. The Hb volumes were divided by the ICV for each participant ($\frac{Hb Volume}{ICV}\times100$) to adjust for individual differences in brain size. According to the results of the ANCOVA, which included age, sex, and education level as covariates, there was a significant difference in the volume ratio of the right Hb between the MDD and healthy control groups (F = 5.544, *P* = 0.022). This is consistent with our initial study results, in which the volume of the right Hb differed significantly between the two groups. The partial eta-squared values (representing the effect size) for the left and right Hb volume ratios were small (η^2^=0.001) and relatively large (η^2^=0.080), respectively. Supplementary Table S1 provides a summary of the ANCOVA results.

**Supplementary Table S1**. Comparison of the normalized habenula volume between the MDD and HC groups, controlling for potential confounders

| Volume Ratio | MDD (n = 33) | | HC (n = 36) | | F | *P* | partial η^2^ |
| --- | --- | --- | --- | --- | --- | --- | --- |
|  | M | SD | M | SD |  |  |  |
| Right Hb | 12.43×10^-4^ | 2.28×10^-4^ | 12.88×10^-4^ | 2.79×10^-4^ | 5.544 | 0.022^*^ | 0.080 |
| Left Hb | 12.75×10^-4^ | 2.26×10^-4^ | 12.87×10^-4^ | 3.52×10^-4^ | 0.037 | 0.848 | 0.001 |
| Statistical analysis was performed using an analysis of covariance. Analyses were adjusted for age, sex, and education level. The normalization of the Hb volumes was performed by dividing the habenula volumes by the ICV for each participant ($\frac{Hb Volume}{ICV}\times100$) to adjust for individual differences in brain size.  ^*^ indicates a significant difference (*P* < 0.05). Abbreviations: HC, healthy control; MDD, major depressive disorder; M, mean; SD, standard deviation; partial η^2^, partial eta-squared; ICV, intracranial volume; Hb, habenula | | | | | | | |

This has been described in the Methods, Results, and Discussion sections as follows:

Methods section (page 14, lines 263–268): “Furthermore, to adjust for potential confounding factors, analysis of covariance (ANCOVA) was conducted comparing the normalized right and left Hb volumes between the MDD and HC groups, controlling for age, sex, education level, and individual differences in brain size. The normalization of the Hb volumes was performed using the total intracranial volume (ICV) from the 3T MRI. The Hb volumes were divided by the ICV for each participant ($\frac{Hb Volume}{ICV}\times100$) to adjust for individual differences in brain size.”

Results section (page 19-20, lines 354-360): “According to the results of the ANCOVA, which included age, sex, and education level as covariates, there was a significant difference in the right Hb volume ratio between the MDD and normal groups (F = 5.544, *p* = 0.022, Supplementary Table S1). This is consistent with the results from the Student’s t-test analysis, in which the volume of the right Hb significantly differed between the two groups. The partial eta-squared values (representing the effect size) for the left and right Hb volume ratios were small (η^2^ = 0.001) and relatively large (η^2^ = 0.080), respectively.”

Discussion section (page 22, lines 377-378): “In addition, in the analysis that included age, sex, and education level as covariates, the right Hb volume ratio was significantly different between the healthy and depressed groups.”

**Comment #12.**

Results: Were patients left or right handed? If mixed this needs to be controlled for in the analysis due to laterality effects.

**Response**: As described in the manuscript, we performed the Edinburgh Handedness Test and included only subjects identified as right-handed, as described in the eligibility criteria.

Handedness is described as follows (page 8, lines 135–136): “The common eligibility criteria are follows: (i) aged 20–65 years; (ii) identified as right-handed using the Edinburgh Handedness Test;”

**Comment #12.**

Line 264: The severity is established according to the rating scale and not the DSM 5. This should be established before the study is conducted and is unusual to be mentioned in the results section. Although you could use the number of symptoms for establishing depression severity, you should not use the DSM 5 because it assumes a dichotomous rather than a continuous approach so it really doesn’t provide a measure of severity, the depression rating scale is usually used.

**Response**: As per your advice, we have added the following in the Methods and Results sections and to Table 1.

Methods section (page 9, lines 161-163): “As per a previous study [41], severity on the Korean version of the HDRS-17 was defined based on the total score as follows: within the normal range (0–6), mild depression (7–17), moderate depression (18–24), and severe depression (≥ 25).”

Results sections (page 16, lines 289–291): “When the severity of depression was classified according to the range of the total score on the HDRS-17, 38 (55%), 14 (20%), 13 (19%), and 4 (6%) were classified as mild, moderate, and severe depression, respectively.”

**Comment #13.**

Discussion: You may want to expand on the limitations. I understand that ROI meta-analyses have not reported the habenula in the past because lack of studies. See for example:

D. Arnone, A.M. McIntosh, K.P. Ebmeier, M.R. Munafò, I.M. Anderson. Magnetic resonance imaging studies in unipolar depression: Systematic review and meta-regression analyses, European Neuropsychopharmacology, Volume 22, Issue 1, 2012, Pages 1-16, ISSN 0924-977X, https://doi.org/10.1016/j.euroneuro.2011.05.003. (<https://www.sciencedirect.com/science/article/pii/S0924977X11001027>)

However, it would seem logical to try to explain why MRI whole brain meta-analyses which used t-maps have not reported abnormalities in this region. Some potential suggestions for discussion depending on whether you included unipolar patients or also bipolar:

Arnone D, Job D, Selvaraj S, Abe O, Amico F, Cheng Y, Colloby SJ, O'Brien JT, Frodl T, Gotlib IH, Ham BJ, Kim MJ, Koolschijn PC, Périco CA, Salvadore G, Thomas AJ, Van Tol MJ, van der Wee NJ, Veltman DJ, Wagner G, McIntosh AM. Computational meta-analysis of statistical parametric maps in major depression. Hum Brain Mapp. 2016 Apr;37(4):1393-404. doi: 10.1002/hbm.23108. Epub 2016 Feb 8. PMID: 26854015; PMCID: PMC6867585.

Stefania Pezzoli, Louise Emsell, Sarah W. Yip, Danai Dima, Panteleimon Giannakopoulos, Mojtaba Zarei, Stefania Tognin, Danilo Arnone, Anthony James, Sven Haller, Sophia Frangou, Guy M. Goodwin, Colm McDonald, Matthew J. Kempton, Meta-analysis of regional white matter volume in bipolar disorder with replication in an independent sample using coordinates, T-maps, and individual MRI data, Neuroscience & Biobehavioral Reviews, Volume 84, 2018, Pages 162-170, ISSN 0149-7634, https://doi.org/10.1016/j.neubiorev.2017.11.005. (https://www.sciencedirect.com/science/article/pii/S0149763417304906)

Wise T, Cleare AJ, Herane A, Young AH, Arnone D. Diagnostic and therapeutic utility of neuroimaging in depression: an overview. Neuropsychiatr Dis Treat. 2014;10:1509-1522. Published 2014 Aug 19. doi:10.2147/NDT.S50156

**Response**: Thank you for your comment. As you mentioned, previous ROI meta-analyses did not include individual studies regarding the habenula, so it was impossible to determine an association between the habenula and depression. In addition, there were four differences between this study and previous studies. First, the subjects of one meta-analysis (Pezzoli et al., 2018) were patients with bipolar depression, while our study focused only on patients with unipolar depressive disorder. Second, the other meta-analyses (Aaron et al., 2012 & 2016) differed in the diagnostic system and scales for diagnosing MDD. Our study diagnosed MDD using the DSM-5 diagnostic system, but the papers in the meta-analyses used other diagnostic systems such as the DSM-III & IV, Schedule for Affective Disorders and Schizophrenia (SADS), Kiddie Schedule for Affective Disorders and Schizophrenia, and ICD-10. Third, the previous studies do not seem to have used structured interviews such as the SCID when selecting subjects. Fourth, the habenula is a structure included in both white and gray matter; however, previous studies were conducted only in white matter or gray matter; therefore, it may have been difficult to find an association between depression and habenula. Fifth, the habenula can be visualized in MRIs from 7.0 Tesla scanners due to improved spatial resolution compared to lower tesla scanners, so the resolution may have been insufficient to clearly delineate the habenula in previous studies that used lower tesla MRIs.

Therefore, we revised the manuscript in the Conclusion section as follows (page 25, lines 452–460): “Previous studies, including meta-analyses, have investigated structural brain abnormalities in depression [65-67]; however, our study is distinct from previous studies. In most previous studies, the Hb were not designated as ROIs, and most studies used lower resolution brain MRIs from 3T, 1.5T, or 1.0T scanners [65, 66]. In addition, the clinical diagnoses of the participants were different (unipolar depression in our study vs. bipolar depression in a previous study [67]), or the diagnostic system or clinical scales [65, 66] used in the studies were dissimilar to ours. Moreover, there were differences in the treatment settings, brain imaging techniques, and methods of obtaining and analyzing the ROIs between the current and previous studies [65-67].”

Given your suggestion, we described the limitations as follows (page 26, lines 477–486): “Although the results of this study are thought to contribute to clarifying the etiology of MDD patients in the future, it is not likely that brain imaging studies can be applied to biomarker for the diagnosis, treatment response, and recurrence of MDD yet [72]. Owing to the relatively small number of subjects in this study, it is difficult to generalize our results. In addition, more advanced neuroimaging analyses and techniques such as machine learning methods should be attempted to further reveal evidence regarding the association between depression and the Hb [73]. Additionally, the type, amount, and duration of psychotropic medications being taken by each subject in the depression group might have affected the outcome. Further replication studies with more control over such variables and larger sample sizes are needed.”
