# Supplementary material for: Left-right asymmetric and smaller right habenula volume in major depressive disorder on high-resolution 7-T magnetic resonance imaging
Source: PLoS One. 2021 Aug 3;16(8):e0255459. doi: 10.1371/journal.pone.0255459 (PMC8330903; doi:10.1371/journal.pone.0255459)
Supplement: Attachment — Submitted filename: Response to Reviewers_R3.docx [file pone.0255459.s004.docx]

**Responses to Reviewer #3:**

We thank the reviewer for their comments regarding our article. We believe that this article has been considerably improved based on the reviewer’s comments and suggestions. Please see below for individual responses to their comments:

**Review Comments to the Author**

I can see that the authors made some changes. The statistical analysis remains a concern.

**Comment #1.**

The self-report questionnaire for personality disorder is not the same as a clinical interview. Please, discuss in the limitations as patients may still have a personality disorder.

**Response**: We agree with your comment. As per your advice, we described the limitations of using the self-report questionnaire (SCID-5-SPQ) to exclude participants with personality disorders as follows:

Limitations section (page 26, lines 490–493): “Due to using self-reported measures, not structured interviews, to screen for personality disorders, there is a possibility of self-report bias. Therefore, we might not have accurately excluded participants with personality disorders.”

**Comment #2.**

Please, specify in your inclusion/exclusion criteria whether patients met criteria for any disorder other than MDD. It is essential to comment on comorbidities. As you know anxiety disorders (and not only) are highly comorbid with MDD.

**Response**: As per your advice, we have added this in the Methods section (page 9, lines 144-153) as follows: “In addition, participants in the MDD group did not have any of the following psychiatric comorbidities: schizophrenia spectrum and other psychotic disorders (delusional disorder, brief psychotic disorder, schizophreniform disorder, schizophrenia, schizoaffective disorder, catatonia); major anxiety disorders (panic disorder, social anxiety disorder, specific phobia); obsessive-compulsive and related disorders (obsessive-compulsive disorder, body dysmorphic disorder, hoarding disorder, trichotillomania, excoriation disorder); or disruptive, impulse-control, and conduct disorders (oppositional defiant disorder, intermittent explosive disorder, conduct disorder). If a patient's psychiatric symptoms could be explained as being due to MDD, we did not specify an additional diagnosis for the patient according to DSM-5.”

**Comment #3.**

How many clinicians assessed the participants? Did you check inter-rater reliability?

**Response**: Only one clinician (SGK) evaluated all the participants. After the initial prescreening, board-certified psychiatrist (SGK) interviewed all participants to assess their eligibility for the present study using the Structured Clinical Interview for Diagnostic and Statistical Manual of Mental Disorders, 5th edition (SCID). In addition, after the participants completed a self-report patient questionnaire for the SCID-5-personality disorder (SCID-5-SPQ), the clinician reviewed it and asked additional questions to exclude any participants with personality disorder.

**Comment #4.**

Need a citation for the Edinburgh test.

**Response**: Thank you for the comment. As per your suggestion, we added the reference for the Edinburgh Inventory to the first sentence of the second paragraph of the Methods section (page 8, line 136) as follows: “The common eligibility criteria are follows: (i) aged 20–65 years; (ii) identified as right-handed using the Edinburgh Handedness Test [40]; (iii) no unstable or major medical or neurological disorders within the past 1 year; (iv) no history of cerebrovascular accident; (v) no history ono substance use disorder within the past 1 year; (vi) no personality disorder, intellectual disability, or neurocognitive disorders; (vii) no current serious suicide risk; (viii) no history of significant brain injury or previous abnormal findings on brain imaging; (ix) no relative or absolute contraindications for MRI (e.g., metal material in the body); and (x) not pregnant or lactating.”

**Reference**

40.	Oldfield R. The assessment and analysis of handedness: The Edinburgh inventory. Neuropsychologica. 1971;9: 97–113.

**Comment #5.**

It appears that paired t tests is still used as the main analysis. This is incorrect in my view as this doesn’t consider the confounders. Please report the results as ANCOVA and please, correct the level of significance for the number of regions you are investigating (2 in this case).

**Response**: We have accommodated your concerns and made the following corrections, as you recommended. For comparisons between the MDD and HC groups, we performed an ANCOVA analysis controlling for confounders rather than conducting a Student's t-test analysis. We also adjusted the significance level to 0.05/2 using the Bonferroni method as you advised, so we only rejected a null hypothesis if the p-value was less than 0.025. We presented the ANCOVA analysis as the main result (Table 2), and the results derived by Student’s t-tests were transferred to the supplementary material. We described this in the Methods and Results sections as follows:

Material and methods section (page 14, line 267–page 15, line 280): “To adjust for potential confounding factors, analysis of covariance (ANCOVA) was conducted comparing the normalized right and left Hb volumes between the MDD and HC groups, controlling for age, sex, education level, and individual differences in brain size. The normalization of the Hb volumes was performed using the total intracranial volume (ICV) from the 3T MRI. The Hb volumes were divided by the ICV for each participant ($\frac{Hb Volume}{ICV}\times100$) to adjust for individual differences in brain size. Since the number of regions we investigated in this analysis was two, the Bonferroni adjusted level of significance (alpha) was calculated to be 2.5%. We rejected the null hypothesis when the p-value was less than the adjusted alpha.

Furthermore, the group difference (MDD vs. HC for volume and T1 value of the right and left Hbs) and the inter-hemispheric difference (right vs. left Hb volume and T1 value) in each group were analyzed using Student’s t-test and paired t-test, respectively. Normality was confirmed using the Kolmogorov-Smirnov method. When not satisfying the homogeneity of variance using Levene’s test, the difference was tested using Welch’s t-test.”

Results section (page 19, line 358–page 20, line 366): “In the between group comparison, according to the results of the ANCOVA, which included age, sex, and education level as covariates, there was a significant difference in the right Hb volume ratio between the MDD and HC groups (F = 5.544, *p* = 0.022, **Table 2**). The partial eta-squared values (representing the effect size) for the left and right Hb volume ratios were small (η^2^ = 0.001) and relatively large (η^2^ = 0.080), respectively. In the analysis using Student’s t-test, participants with MDD had a significantly smaller right Hb volume than those in the HC group (*P* = 0.018, **Supplementary table S1**). There was no significant difference in the left Hb volume between the two groups. This is consistent with the results from the ANCOVA, in which the volume of the right Hb differed significantly between the two groups.”

**Table 2. Comparison of the normalized habenula volume between the MDD and HC groups, controlling for potential confounders**

| Volume Ratio | MDD (n = 33) | | HC (n = 36) | | F | *P* | partial η^2^ |
| --- | --- | --- | --- | --- | --- | --- | --- |
|  | M | SD | M | SD |  |  |  |
| Right Hb | 12.43×10^-4^ | 2.28×10^-4^ | 12.88×10^-4^ | 2.79×10^-4^ | 5.544 | 0.022^*^ | 0.080 |
| Left Hb | 12.75×10^-4^ | 2.26×10^-4^ | 12.87×10^-4^ | 3.52×10^-4^ | 0.037 | 0.848 | 0.001 |
| Statistical analysis was performed using an analysis of covariance. Analyses were adjusted for age, sex, and education level. The normalization of the Hb volumes was performed by dividing the habenula volumes by the ICV for each participant ($\frac{Hb Volume}{ICV}\times100$) to adjust for individual differences in brain size.  ^*^ indicates a significant difference (*P* < 0.025).  Abbreviations: HC, healthy control; MDD, major depressive disorder; M, mean; SD, standard deviation; partial η^2^, partial eta-squared; ICV, intracranial volume; Hb, habenula | | | | | | | |

**Supplementary Table S1**. **Group differences in the volume and T1 value of the habenula segmented by both examiners** **between MDD and HC**

|  | Hemisphere | MDD (n = 33) | HC (n = 36) | t score | *P* values |
| --- | --- | --- | --- | --- | --- |
| Volume  (in mm^3^ ± SD) | Right | 16.43 ±3.24 | 18.54 ± 3.91 | −2.431 | 0.018^*^ |
|  | Left | 18.37 ± 3.39 | 18.47 ± 5.04 | −0.090 | 0.929 |
| T1  (value ± SD) | Right | 1200.96 ± 41.29 | 1207.84 ± 32.35 | −0.773 | 0.442 |
|  | Left | 1225.03 ± 34.81 | 1220.51 ± 31.90 | 0.564 | 0.575 |

^*^ indicates significant difference (*P* < 0.05).
The statistical analysis was performed using a Student’s t-test
Abbreviations: HC, healthy control; MDD, major depressive disorder; SD, standard deviation

**Comment #6.**

The majority of the patients suffered a mild form of illness. This is a major limitation because how representative of MDD is mild illness? The majority of the studies investigate moderate to severe levels of illness. My suggestion is to discuss this in the limitation as it might at least contribute to explain some of your findings.

**Response**: As you mentioned, 42% (n = 14) of patients with MDD had mild depression based on the HDRS score on the MRI scanning day. However, it is presumed that this is because the MDD group had been continuously receiving treatment, such as medication and psychotherapy. In fact, 51% of the patients with MDD had recurrence (n = 15) and remission (n = 2) of major depressive episodes. In general, the state of depression is not directly related to the structural state of the brain. However, as you suggested, since the severity of depressive symptoms was different, this might have affected the outcomes of this study. Therefore, we have described this in the Limitations section It now reads as follows (page 27, lines 500–504): “Additionally, the severity of MDD symptoms and the type, amount, and duration of psychotropic medications being taken by each participant in the MDD group might have affected the outcomes. In future research, it is essential to compare subdivided brain regions according to the patients’ severity of symptoms, whether or not they are taking any medications, and medication type.”

**Comment #7.**

Did any patient used more than one psychotropic medication? Did anyone use anything else other than antidepressants? The use of psychotropics is a major limitation.

**Response**: As per your suggestion, we have described the use of psychotropics in participants in the MDD group. We have described this in the Results and Limitations sections as follows:

Result section, page 17, line 323–page 18, line 326: “In the MDD group, other psychotropic drugs were being taken as follows: benzodiazepine (n = 17), zolpidem (n = 2), quetiapine (n = 7), aripiprazole (n = 6), and olanzapine (n = 1). Second-generation antipsychotics were prescribed as adjunctive treatments for MDD.”

**Table 1. Demographic and clinical characteristics of the MDD and HC groups**

| Clinical variables | MDD (n = 33) | HC (n = 36) | *P* values |
| --- | --- | --- | --- |
| Age at scan, years (mean ± SD) | 40.58 ± 14.16 | 35.17 ± 12.00 | 0.093^a^ |
| Sex (male : female) | 8 : 25 | 12 : 24 | 0.406^b^ |
| Education, years (mean ± SD) | 12.73 ± 3.45 | 15.22 ± 1.91 | 0.001^a^ |
| Severity ranges for the HDRS-17 score, n (%) |  |  |  |
| Within the normal range (0–6) | 2 (6) | 36 (100) | N/A |
| Mild depression (7­–17) | 14 (42) | 0 | N/A |
| Moderate depression (18–24) | 13 (39) | 0 | N/A |
| Severe depression (≥ 25) | 4 (12) | 0 | N/A |
| The number of episodes (mean ± SD) | 2.58 ± 1.39 | N/A | N/A |
| Duration of current depressive episode, weeks (mean ± SD) | 73.93 ± 16.12 | N/A | N/A |
| Duration of illness, months (mean ± SD) | 63.45 ± 57.45 | N/A | N/A |
| The use of psychotropic drugs, n (%) |  |  |  |
| Antidepressants | 27 (82) | N/A | N/A |
| Benzodiazepine | 19 (58) | N/A | N/A |
| Second-generation antipsychotics | 14 (42) | N/A | N/A |
| Duration of antidepressant use, weeks (mean ± SD) | 82.18 ± 26.24 | N/A | N/A |
| MDD subgroups^c^, n (%) |  |  |  |
| First episode | 12 (36) | N/A | N/A |
| Treatment-resistance | 6 (18) | N/A | N/A |
| Recurrence | 15 (45) | N/A | N/A |
| Remission | 2 (6) | N/A | N/A |
| HDRS-17 score (mean ± SD) | 15.95 ± 5.40 | 2.53 ± 2.41 | < 0.001^a^ |
| BHS score (mean ± SD) | 12.52 ± 5.93 | 1.19 ± 1.47 | < 0.001^a^ |
| BDI score (mean ± SD) | 27.79 ± 12.66 | 3.50 ± 3.68 | < 0.001^a^ |
| CGI score (mean ± SD) | 4.03 ± 0.95 | 1.03 ± 0.17 | < 0.001^a^ |
| SSI score (mean ± SD) | 14.00 ± 8.69 | 1.00 ± 1.53 | < 0.001^a^ |

^a^ Student’s t-test; ^b^ Chi-square test; ^c^ Some patients belonged to more than one group.

Data are presented as means ± standard deviations or numbers (percentages) unless otherwise indicated.

Abbreviations: BAI, Beck Anxiety Inventory; BDI, Beck depression inventory; BHS, Beck Hopelessness Scale; CGI, clinical global impression scale; HDRS, Hamilton Depression Rating Scale; HC, Healthy Control; MDD, Major Depressive Disorder; SD, Standard Deviation; SSI, Scale for Suicide Ideation.

Limitations section, page 27, lines 500–505: “Additionally, the severity of MDD symptoms and the type, amount, and duration of psychotropic medications being taken by each participant in the MDD group might have affected the outcomes. In future research, it is essential to compare subdivided brain regions according to the patients’ severity of symptoms, whether or not they are taking any medications, and medication type. Further replication studies with more control over such variables and larger sample sizes are needed.”

”

**Comment #8.**

I don’t understand the results. Why are you still referring to paired t tests? This approach is not correct as it does not consider all the confounders and individual brain differences. Please, use ANCOVA throughout and amend table 2 and 3 which are still referring to paired t test. Really confusing.

**Response**: We have accommodated your concerns and made the following corrections, as we also noted in our response to Comment #5. For comparisons between the MDD and HC groups, we performed an ANCOVA analysis controlling for confounders rather than conducting a Student's t-test analysis. We also adjusted the significance level to 0.05/2 using the Bonferroni method as you advised, so we only rejected a null hypothesis if the p-value was less than 0.025. We presented the ANCOVA analysis as the main result (Table 2), and the results derived by Student’s t-tests were transferred to the supplementary material. We described this in the Methods and Results sections as follows:

Material and methods section (page 14, line 267–page 15, line 280): “To adjust for potential confounding factors, analysis of covariance (ANCOVA) was conducted comparing the normalized right and left Hb volumes between the MDD and HC groups, controlling for age, sex, education level, and individual differences in brain size. The normalization of the Hb volumes was performed using the total intracranial volume (ICV) from the 3T MRI. The Hb volumes were divided by the ICV for each participant ($\frac{Hb Volume}{ICV}\times100$) to adjust for individual differences in brain size. Since the number of regions we investigated in this analysis was two, the Bonferroni adjusted level of significance (alpha) was calculated to be 2.5%. We rejected the null hypothesis when the p-value was less than the adjusted alpha.

Furthermore, the group difference (MDD vs. HC for volume and T1 value of the right and left Hbs) and the inter-hemispheric difference (right vs. left Hb volume and T1 value) in each group were analyzed using Student’s t-test and paired t-test, respectively. Normality was confirmed using the Kolmogorov-Smirnov method. When not satisfying the homogeneity of variance using Levene’s test, the difference was tested using Welch’s t-test.”

Results section (page 19, line 357–page 20, line 366): “In the between group comparison, according to the results of the ANCOVA, which included age, sex, and education level as covariates, there was a significant difference in the right Hb volume ratio between the MDD and HC groups (F = 5.544, *p* = 0.022, **Table 2**). The partial eta-squared values (representing the effect size) for the left and right Hb volume ratios were small (η^2^ = 0.001) and relatively large (η^2^ = 0.080), respectively. In the analysis using Student’s t-test, participants with MDD had a significantly smaller right Hb volume than those in the HC group (*P* = 0.018, **Supplementary table S1**). There was no significant difference in the left Hb volume between the two groups. This is consistent with the results from the ANCOVA, in which the volume of the right Hb differed significantly between the two groups.”

**Table 2. Comparison of the normalized habenula volume between the MDD and HC groups, controlling for potential confounders**

| Volume Ratio | MDD (n = 33) | | HC (n = 36) | | F | *P* | partial η^2^ |
| --- | --- | --- | --- | --- | --- | --- | --- |
|  | M | SD | M | SD |  |  |  |
| Right Hb | 12.43×10^-4^ | 2.28×10^-4^ | 12.88×10^-4^ | 2.79×10^-4^ | 5.544 | 0.022^*^ | 0.080 |
| Left Hb | 12.75×10^-4^ | 2.26×10^-4^ | 12.87×10^-4^ | 3.52×10^-4^ | 0.037 | 0.848 | 0.001 |
| Statistical analysis was performed using an analysis of covariance. Analyses were adjusted for age, sex, and education level. The normalization of the Hb volumes was performed by dividing the habenula volumes by the ICV for each participant ($\frac{Hb Volume}{ICV}\times100$) to adjust for individual differences in brain size.  ^*^ indicates a significant difference (*P* < 0.025).  Abbreviations: HC, healthy control; MDD, major depressive disorder; M, mean; SD, standard deviation; partial η^2^, partial eta-squared; ICV, intracranial volume; Hb, habenula | | | | | | | |

**Supplementary Table S1**. **Group differences in the volume and T1 value of the habenula segmented by both examiners between MDD and HC**

|  | Hemisphere | MDD (n = 33) | HC (n = 36) | t score | *P* values |
| --- | --- | --- | --- | --- | --- |
| Volume  (in mm^3^ ± SD) | Right | 16.43 ±3.24 | 18.54 ± 3.91 | −2.431 | 0.018^*^ |
|  | Left | 18.37 ± 3.39 | 18.47 ± 5.04 | −0.090 | 0.929 |
| T1  (value ± SD) | Right | 1200.96 ± 41.29 | 1207.84 ± 32.35 | −0.773 | 0.442 |
|  | Left | 1225.03 ± 34.81 | 1220.51 ± 31.90 | 0.564 | 0.575 |

^*^ indicates significant difference (*P* < 0.05).
The statistical analysis was performed using a Student’s t-test
Abbreviations: HC, healthy control; MDD, major depressive disorder; SD, standard deviation

However, it does not seem appropriate to use ANCOVA when analyzing inter-hemispheric differences within the same group. ANOVA is a method used to compare the mean of the dependent variable between two or more different groups. Therefore, we performed a paired t-test to compare the mean values of the left and right areas within the same group.

**Comment #9.**

This study has several limitations. I cannot see that limitations were addressed in the discussion. All the points mentioned here and in the previous round of comments including medication status, severity of illness, comorbidities, etc., could be a starting point.

**Response**: As per your suggestion, we separated the Limitation section from the Discussion section and described these issues as limitations as follows (page 26, line 487–page 27, line 505):

**“Limitations**

Although the results of this study are thought to contribute to clarifying the etiology of MDD patients in the future, it is not likely that brain imaging studies can be applied to biomarker for the diagnosis, treatment response, and recurrence of MDD yet [72]. Due to using self-reported measures, not structured interviews, to screen for personality disorders, there is a possibility of self-report bias. Therefore, we might not have accurately excluded participants with personality disorders. In addition, although we attempted to exclude participants with psychiatric disorders other than MDD under the DSM-5, it would have been difficult to entirely exclude individuals who might have also had these other disorders. Thus, there is a possibility that structural changes in the brain were caused by comorbid disorders. Owing to the relatively small number of subjects in this study, it is difficult to generalize our results. In addition, more advanced neuroimaging analyses and techniques such as machine learning methods should be attempted to further reveal evidence regarding the association between depression and the Hb [73]. Additionally, the severity of MDD symptoms and the type, amount, and duration of psychotropic medications being taken by each participant in the MDD group might have affected the outcomes. In future research, it is essential to compare subdivided brain regions according to the patients’ severity of symptoms, whether or not they are taking any medications, and medication type. Further replication studies with more control over such variables and larger sample sizes are needed.”
